# Yeast ribosomal protein L7 and its homologue Rlp7 are simultaneously present at distinct sites on pre-60S ribosomal particles

**DOI:** 10.1093/nar/gkt726

**Published:** 2013-08-13

**Authors:** Reyes Babiano, Gwenael Badis, Cosmin Saveanu, Abdelkader Namane, Antonia Doyen, Antonio Díaz-Quintana, Alain Jacquier, Micheline Fromont-Racine, Jesús de la Cruz

**Affiliations:** ^1^Departamento de Genética, Universidad de Sevilla, E-41012 Seville, Spain, ^2^Institut Pasteur, Génétique des Interactions Macromoléculaires, CNRS UMR-3525, Paris, France and ^3^Instituto de Bioquímica Vegetal y Fotosíntesis, Universidad de Sevilla-CSIC, Seville, Spain

## Abstract

Ribosome biogenesis requires >300 assembly factors in *Saccharomyces cerevisiae*. Ribosome assembly factors Imp3, Mrt4, Rlp7 and Rlp24 have sequence similarity to ribosomal proteins S9, P0, L7 and L24, suggesting that these pre-ribosomal factors could be placeholders that prevent premature assembly of the corresponding ribosomal proteins to nascent ribosomes. However, we found L7 to be a highly specific component of Rlp7-associated complexes, revealing that the two proteins can bind simultaneously to pre-ribosomal particles. Cross-linking and cDNA analysis experiments showed that Rlp7 binds to the ITS2 region of 27S pre-rRNAs, at two sites, in helix III and in a region adjacent to the pre-rRNA processing sites C_1_ and E. However, L7 binds to mature 25S and 5S rRNAs and cross-linked predominantly to helix ES7^L^b within 25S rRNA. Thus, despite their predicted structural similarity, our data show that Rlp7 and L7 clearly bind at different positions on the same pre-60S particles. Our results also suggest that Rlp7 facilitates the formation of the hairpin structure of ITS2 during 60S ribosomal subunit maturation.

## INTRODUCTION

In eukaryotes, ribosome biogenesis is a complex multi-step and multi-component process, which occurs primarily in the nucleolus, although late steps occur in the nucleoplasm and in the cytoplasm [for reviews, see (1,2)] Most of our knowledge concerning ribosome biogenesis in eukaryotes derives from studies with *Saccharomyces cerevisiae*. In the yeast nucleolus, the mature 18S, 5.8S and 25S rRNAs are co-transcribed by RNA polymerase I as a single large precursor rRNA (pre-rRNA) that undergoes co- or post-transcriptional processing, whereas the pre-5S is independently transcribed by RNA polymerase III (Supplementary Figure S1). Pre-rRNA processing occurs concomitantly to most rRNA modification reactions, folding of pre-rRNAs and assembly of most ribosomal proteins (r-proteins) to form pre-ribosomal particles (Supplementary Figure S2). Pre-ribosomal particles contain, in addition to pre-rRNAs and r-proteins, non-ribosomal *trans*-acting factors (1).

In yeast, roughly 300 protein *trans*-acting factors, involved in ribosome biogenesis, have been identified (3,4). These factors likely confer speed, accuracy and directionality to the ribosome synthesis process. The precise mechanisms by which protein *trans*-acting factors operate are still largely unknown. The use of affinity purification combined with quantitative mass spectrometry techniques like isobaric Tag for Relative and Absolute Quantification or SILAC (Stable Isotope Labelling with Amino-acids in Cell culture) allow to measure the timing of binding to and dissociation from pre-ribosomal particles for many protein *trans*-acting factors [e.g., (5,6)]. To better understand the role of these factors in ribosome biogenesis, other experimental approaches have been developed, among them, *in vivo* cross-linking and cDNA analysis (CRAC). This technique allows the identification of the interaction sites between several protein *trans*-acting factors and pre-rRNAs or snoRNAs [e.g. (7–11)].

Ribosomal proteins are active players in the maturation and the nucleo-cytoplasmic transport of pre-ribosomal particles. As for *trans*-acting factors, functional analyses have revealed how loss-of-function mutations in r-protein genes negatively impact on pre-rRNA processing and pre-ribosomal particles transport [e.g. (12–15)]. Little is known about the specific role of r-proteins in driving formation or re-arrangement of structures within pre-ribosomal particles [e.g. (6)]. Moreover, the course of assembly of the r-proteins remains unclear, especially for r-proteins of the large r-subunit [(16–18) and references therein].

A set of *trans*-acting factors, similar to selected r-proteins throughout their entire primary sequence, provides interesting insight into the assembly process and especially into the evolution of ribosome assembly factors and r-proteins. Amongst them are Rlp7, which is paralogous to L7 [L30 in the Yusupov’s nomenclature (19)], as is Rlp24 to L24 (L24e), Mrt4 to P0 and Imp3 to S9 (S4) (20–24). Considering the high degree of homology found, it has been proposed that these factors and their r-protein counterpart successively bind the same rRNA structure, but although the factor binds to the rRNA site within a pre-ribosomal particle, the r-protein binds the same site within the mature r-subunit [discussed in (20,25)]. However, although we have experimentally demonstrated this ‘placeholder hypothesis’ for the relationship between Mrt4 and P0 (23), no validation has been shown for other paralogous pairs. In this work, we have studied the relationship between Rlp7 and L7 at different levels. We report distinct rRNA binding sites for Rlp7 and L7 and co-existence of the two proteins on the same pre-ribosomal particles. Our findings clearly show that the placeholder hypothesis is far from being a rule in ribosome biogenesis and provides insights into the molecular role of Rlp7 during 60S r-subunit assembly.

## MATERIALS AND METHODS

### Strains and microbiological methods

The yeast strains used in this study, which were derivatives of BY4741 or BMA64-1B, are listed in Supplementary Table S1. Most strains were generated by standard recombination techniques or by genetic crosses followed by sporulation, tetrad dissection and phenotypic analysis. All strains were checked by PCR and, when possible, by western blotting. Growth and handling of yeast and standard media were performed by established procedures (26). Yeast cells were grown at 30°C in rich or minimal medium containing either 2% galactose (YPGal, SGal), 2% glucose (YPD, SD) or in minimal medium containing 2% raffinose (SRaf).

### Plasmid constructions

Plasmids are listed in Supplementary Table S2. To generate YCplac111-RLP7-HA, a 1.6 kb PCR product containing the *RLP7* ORF lacking the termination codon and an additional 1 kb upstream the ORF was cloned into pHAC111 (27). The structure of the resulting plasmid was verified by DNA sequencing. This construct complemented the growth of strains harbouring the *GAL::RLP7* allele to the wild-type extent in glucose-containing media.

### Purification of complexes for SILAC quantification and SILAC data analysis

Cells from untagged and Rlp7-TAP tagged strains were grown in minimal medium in presence of either labelled l-lysine-^13^C_6_,^15^N_2_ (Sigma-Aldrich) or regular l-lysine, respectively (50 mg/l). Cell pellets from 1 l of each culture at 1.5 OD_600_ per ml were suspended in lysis buffer, mixed and broken with a French Press. One-step purification for SILAC experiments was performed using magnetic beads coated with immunoglobulin G (IgG) (Life Technologies) as described in (28,29). Proteins were identified by LC-MS/MS on a LTQ-Orbitrap velos instrument (Thermo Fisher Scientific, Bremen) as described in (28). Briefly, protein samples were treated with Endoprotease Lys-C and Trypsin. Digested peptides were desalted and then analysed by LC-MS/MS on a LTQ-Orbitrap velos instrument (Thermo Fisher Scientific, Bremen) (28). Raw MS data from the LTQ-Orbitrap were analysed using the MaxQuant software version 1.3.0.5 (30,31), supported by Andromeda (32) applying a false-discovery rate for both peptide and protein identification at *P* ≤ 0.01. MS/MS spectra were searched against a concatenated *S. cerevisiae* decoy database from UniprotKB. Two missed cleavage were allowed, and only peptides with a minimum of seven amino acids were considered for identification. After data processing, SILAC quantification (H/L ratios) values from the ‘proteinGroups.txt’ output file of MaxQuant were taken for further analysis. The protein list was filtered to remove contaminants and reversed sequences. Proteins with a minimum of two measurements (ratio count ≥ 2) were selected for further analysis, and the H/L ratios were log_2_ transformed. The data are shown as a Supplementary Excel Table (Supplementary Data Set S1).

### Affinity purifications

One-step purification of HTP- and TAP-tagged or untagged cells was performed with IgG-Sepharose beads. About 100 ml of HTP/TAP-tagged or untagged negative control cells were grown to an OD_600_ of 0.8, washed with cold water, harvested and concentrated in 500 µl of ice-cold TNM150 lysis buffer [50 mM Tris (pH 7.8), 1.5 mM MgCl_2_, 150 mM NaCl, 0.1% NP-40, 5 mM β-mercaptoethanol] containing a protease inhibitor cocktail (Complete, Roche). Cells were disrupted by vigorous shaking with glass beads in a Fastprep®-24 (MP Biomedicals) at 4°C, and total cell extracts were obtained by centrifugation in a microcentrifuge at the maximun speed (ca. 16 100× *g*) for 15 min at 4°C. Each supernatant obtained was mixed with 50 µl of IgG-Sepharose beads (GE-Healthcare), previously equilibrated with the TNM150 buffer, and incubated for 2 h at 4°C with end-over-end tube rotation. After incubation, the beads were extensively washed 10 times with 1 ml of the same buffer at 4°C and finally collected. Protein was extracted with Laemmli buffer from both whole-cell extracts and 1/10th of the beads. Proteins were analysed by western blotting using Peroxidase anti-peroxidase soluble complex (Sigma). RNA was extracted from total cell extracts, and the rest of the beads as described in (33,34) and analysed by northern blotting. The oligonucleotides used for northern blot hybridizations are described in the Supplementary Table S3.

### Western blotting analyses and antibodies

Proteins were separated by SDS–PAGE and transferred onto nitrocellulose membranes by standard procedures. The following primary antibodies were used: Peroxidase anti-peroxidase at a dilution of 1:10000, rabbit polyclonal anti-L1 (1:10000; gift from F. Lacroute) (35), rabbit polyclonal anti-L35 (1:5000; gift from M. Seedorf) (36), rabbit polyclonal anti-Has1 (1:5000) (37), mouse monoclonal anti-Nop1 (1:5000; MCA28F2, EnCor Biotechnology) and mouse monoclonal anti-HA (1:5000, Roche). Secondary goat anti-rabbit or anti-mouse horseradish peroxidase-conjugated antibodies (Bio-Rad) were used at a dilution of 1:5000. Proteins were detected using an enhanced chemiluminescence detection kit (Super-Signal West Pico, Pierce).

### CRAC and sequence analysis

*In vivo* CRAC experiments were performed as described previously (9). Briefly, cells expressing HTP-tagged Rlp7 and a non-tagged negative control strain were ultraviolet irradiated; cell extracts were then performed and subjected to a first affinity purification on IgG-Sepharose beads. Purified complexes were partially RNase-digested and subjected to a second affinity purification step on a nickel column under denaturing conditions. RNA molecules cross-linked to Rlp7-HTP were ligated to linkers, amplify by RT-PCR and subjected to Solexa sequencing to identify the relative location along the rDNA of the recovered RNA pieces. Similar experiments were performed with HTP-tagged L7 strains. Data analyses were done with the Integrative Genomics Viewer software (38).

### *In silico* analysis of ribosome structure

Atomic coordinates of the yeast 60S r-subunit were retrieved from the Protein Data Bank (PDB; www.rcsb.org) with the accesion number 3U5D and 3U5E (19). L7 structure was substracted from the 3U5E file (chain F). Rlp7 structure was built with the Modeller 9v2 program (39) using as templates the coordinates provided in pdb files 2ZKR, 3IZR, 3JYW, 3U5E, 3U5E, 3U5I, 3ZF7, 4A1C, 4A1E and 4B6A. Sequence identities percentages of the target versus the templates ranged from 42 (2ZKR) to 48% (3IZR). All models were visualized with the UCSF Chimera program (40). Secondary structure of pre- and mature rRNAs were taken from Granneman *et al.* (7) and The Comparative RNA Web Site (http://www.rna.icmb.utexas.edu/), respectively.

## RESULTS

### Overlapping pattern of Rlp7 and L7 association with pre-ribosomal particles

Yeast r-protein L7 shares a notable sequence similarity with the *trans*-acting factor Rlp7 throughout its entire primary sequence (41) (Supplementary Figure S3). Given this close homology, we could even model the predicted structure of most of the Rlp7 protein (from Lys_84_ to Asn_322_) on the basis of the structure of L7 in the crystal of yeast 60S r-subunit (19). The N-terminal part of Rlp7 (from Met_1_ to Asp_83_) shows a high probability of being intrinsically disordered. This is also the case for the N-terminal region of L7 r-protein (from Met_1_ to Lys_21_) (A.D.-Q., unpublished results). As expected, both core structures satisfactorily superimposed (Supplementary Figure S4). Taking into account the degree of homology and the predicted structural resemblance, we aimed to test whether Rlp7 and L7 could exchange at a particular stage of the 60S r-subunit maturation pathway.

**Figure 1.**
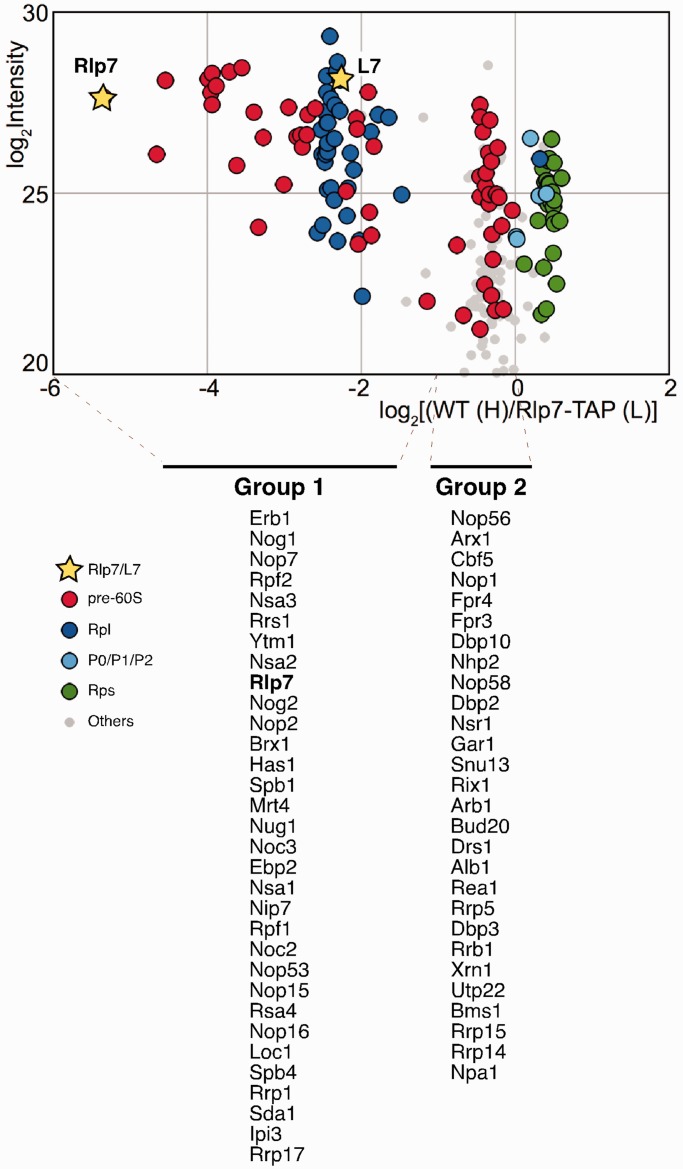
Rlp7-associated pre-ribosomal particles contain the L7 r-protein. Wild-type and Rlp7-TAP cells were mixed in equal proportions prior to complex purification. The log_2_ of SILAC ratios (median value of Wild-type/Rlp7-TAP peptide ratio) were plotted against the sum of the intensity of all the peptides for each protein. Dots are coloured according to protein function: pre-60S factors (red), 60S r-proteins (Rpl, blue), r-stalk proteins (P0/P1/P2, light blue), 40S r-proteins (Rps, green) and proteins of other different functions (grey). Yellow stars indicate the Rlp7 and L7 values. The identity of pre-60S factors specifically enriched (Group 1) or not (Group 2) is indicated below the graph. These factors are listed from their highest to lowest intensity values (see Supplementary Data Set S1).

To determine whether Rlp7 and L7 co-exist in the same pre-ribosomal particles, we performed a SILAC experiment combined with LC-MS/MS mass spectrometry to monitor the relative amount of L7 present in affinity purified pre-ribosomal complexes from a strain expressing TAP-tagged Rlp7. We have identified 60 pre-60S factors and most r-proteins from 60S (Rpl) and 40S (Rps) r-subunits (Figure 1 and Supplementary Data Set S1). Proteins clearly arranged in two groups. Group 1 (specifically enriched proteins; log_2_ of SILAC ratio between −6 and −1) comprises most Rpl proteins (including L7) and 31 pre-60S factors, in addition to Rlp7, which were strongly enriched in the Rlp7-TAP associated sample; among them, early-acting pre-60S factors including several A_3_ factors (Erb1, Nop7, Nsa3, Ytm1 and Nop15) (6) were specifically abundant as judged from the total MS signal intensity. Group 2 (non-specific proteins; log_2_ of SILAC ratio between −1 and 0) includes 28 pre-60S factors, among them late-acting pre-60S factors (e.g. Arb1, Bud20, Arx1 and Alb1) and components of snoRNPs (e.g. Nop56, Cbf5, Nop1, Nhp2, Nop58, Gar1 and Snu13). Interestingly, all detected Rps proteins, the Rpl proteins from the r-stalk (P0, P1 and P2) and L10 appeared at values considered as contamination (log_2_ of SILAC ratio > 0). These results are consistent with the fact that the assembly of the r-stalk proteins and L10 into 60S r-subunits occurs predominantly in the cytoplasm (42,43). These results, confirmed by a second independent SILAC experiment, clearly identify Rlp7 as an early pre-60S assembly factor and strongly suggest that Rlp7 and L7 bind to the same pre-ribosomal particles (Supplementary Data Set S1).

**Figure 2.**
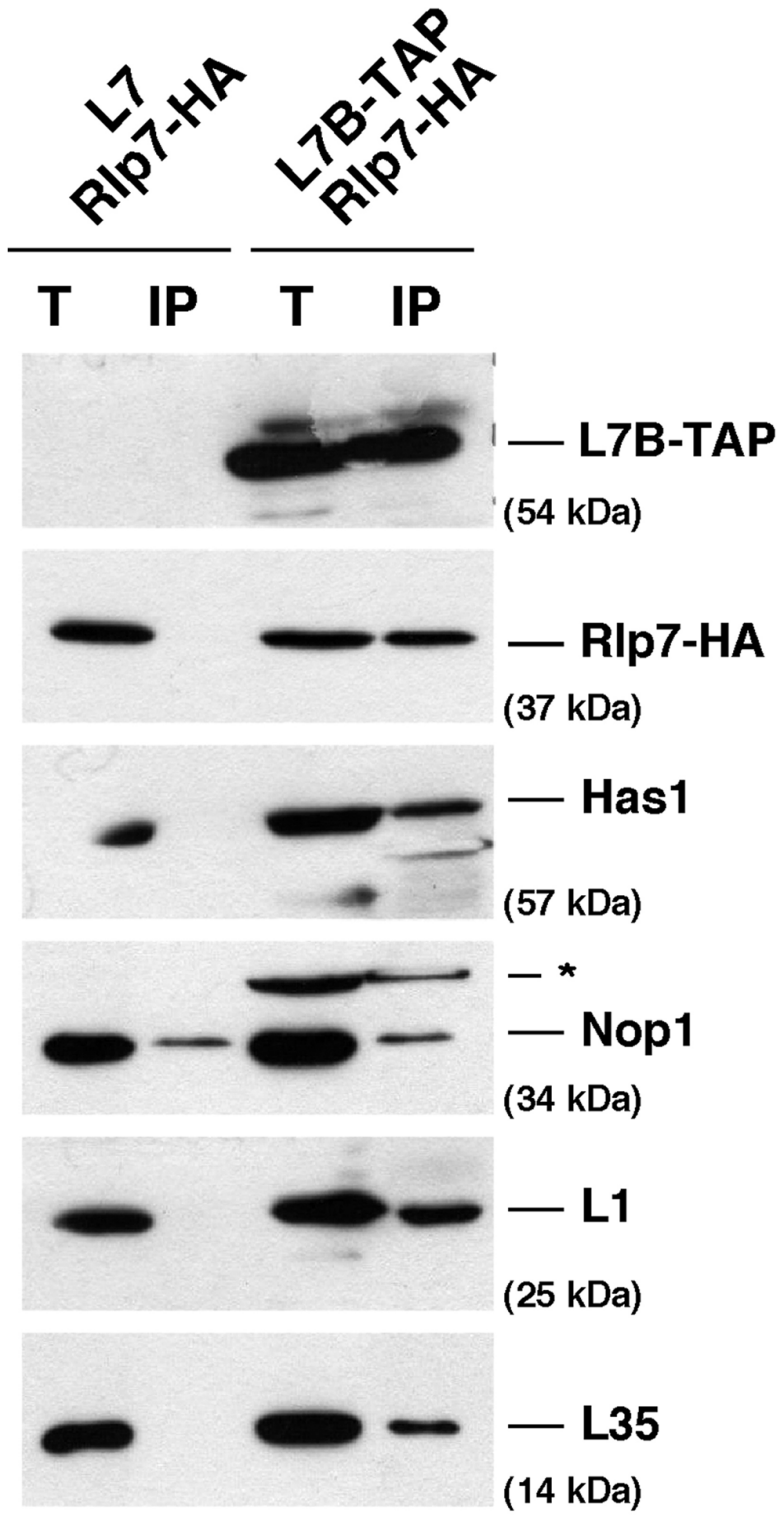
Rlp7 and L7 association with pre-ribosomal particles is not mutually exclusive. Extracts were prepared from cells co-expressing Rlp7-HA and L7B-TAP or, as a control, Rlp7-HA and untagged L7B. Total extracts (T) and L7B-TAP affinity-purified samples (IP) were analysed by western blotting. Co-precipitation of Rlp7-HA, Has1, Nop1 and r-proteins L1 and L35 was tested with specific antibodies. The asterisk corresponds to a L7B-TAP cross-reaction.

To confirm these results, we performed IgG-Sepharose purification with extracts of cells expressing both C-terminal TAP-tagged L7B and HA-tagged Rlp7 proteins. Analysis of purified complexes by SDS–PAGE and western blotting demonstrated the association of Rlp7-HA with L7B-TAP, the r-proteins L1 and L35 and the *trans*-acting factor Has1 (Figure 2). This association seems to be specific, as no co-purification was observed when a strain harbouring a non-tagged L7B was used as a control. In contrast, the *trans*-acting factor Nop1 did not associate with L7B-TAP (Figure 2). This result correlates well with the identification of Has1 in Group 1 and Nop1 in Group 2 of Rlp7-TAP associated proteins. We conclude that both Rlp7 and L7 are able to simultaneously bind to the same particles.

**Figure 3.**
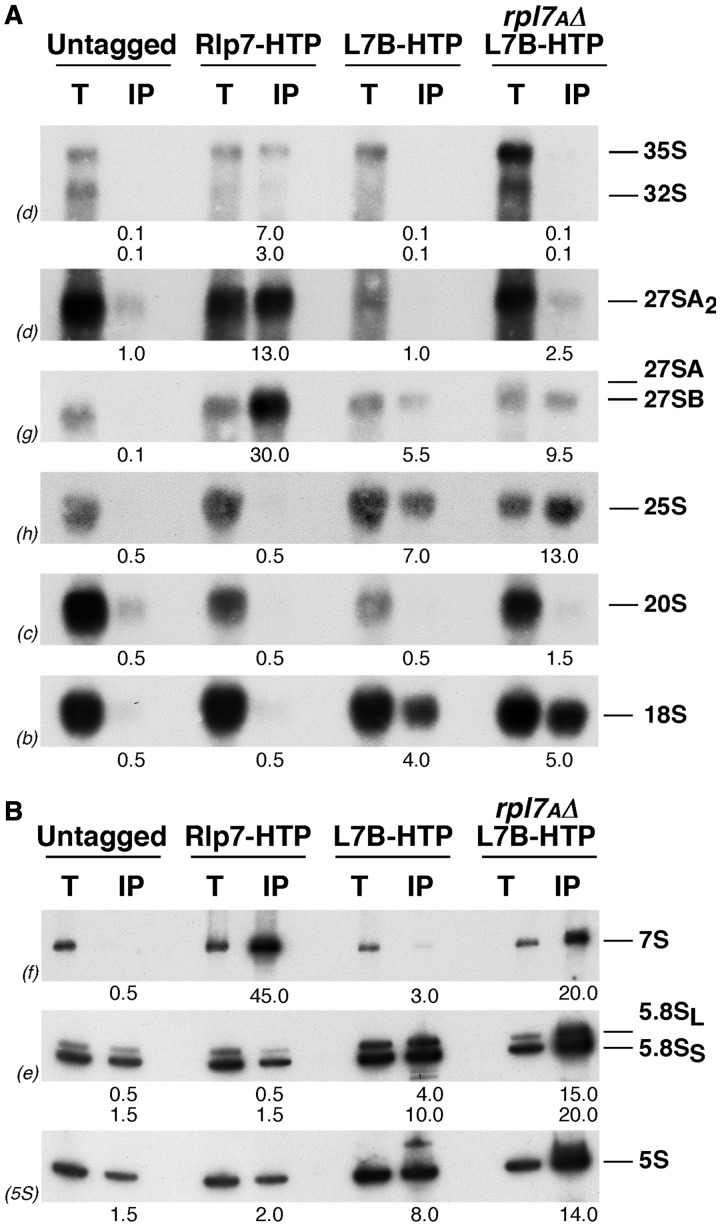
Association of Rlp7 and L7 with r-particles. HTP-tagged Rlp7 and L7 were affinity-purified from extracts of the indicated strains. RNA was isolated from total extract (T) and purified samples (IP) and analysed by northern blotting. (**A**) Large pre- and mature rRNAs. (**B**) Small pre- and mature rRNAs. Probes (in parentheses) are described in Supplementary Figure S1. Signal intensity was measured by phosphorimager scanning; values (below each IP lane) refer to the percentage of each RNA recovered after purification.

Rlp7 has been shown to localize in the nucleolus (20,21) and associate to pre-60S r-particles (6,21). To further characterize Rlp7-containing pre-ribosomal particles, we performed a one-step IgG-Sepharose purification with lysates from a C-terminal Rlp7-HTP (His_6_-TEV-Protein A)-tagged strain and a non-tagged negative control and identified the associated pre-rRNA species. As shown in Figure 3, there was a significant enrichment for the 27SA_2_, 27SB and 7S pre-rRNAs and a modest enrichment for 35S pre-rRNA for the Rlp7-HTP-precipitated preparations. No enrichment over the background levels was detected for mature rRNAs. These results indicate that Rlp7 is a stable component of early and medium pre-60S particles that dissociates from particles following 7S pre-rRNA processing. To explore L7 timing of assembly, we performed the reverse experiment by affinity purification of L7B-HTP containing complexes from C-terminal HTP-tagged L7B strain. As shown in Figure 3, and as expected for a 60S r-protein [e.g. (14,18)], there was significant co-purification of mature rRNAs with L7B-HTP. Precursors 27S and 7S pre-rRNAs were clearly also detected, in contrast to 35S and 20S pre-rRNAs that were found at the background level (Figure 3). The contribution of the *RPL7A* gene to growth and ribosome biogenesis is more important than that of *RPL7B* (15) (Supplementary Figure S5); thus, to improve the pre-rRNA co-precipitation by L7B-HTP, we made use of an isogenic HTP-tagged strain disrupted for the *RPL7A* gene. As expected, this strain displayed a slow-growth phenotype (Supplementary Figure S5). As also shown in Figure 3, L7B-HTP more efficiently co-precipitated pre- and mature rRNAs, especially 7S pre-rRNA, in this genetic background. Thus, L7 stably assembles into early and medium pre-60S particles. Consistently, when we monitor the localization of a functional L7B-GFP construct on induction of the dominant negative *NMD3Δ100* allele, which lead to the retention of pre-60S r-particles in the nucle(ol)us (44), this accumulates in the nucle(ol)us from most cells examined (Supplementary Figure S6), similarly as does a L3-GFP reporter used as positive control for nucle(ol)ar assembly.

Taken together, these data demonstrate that Rlp7 and L7 are present in similar pre-ribosomal complexes at the same time in the nuclear stages of 60S r-subunit assembly. This conclusion is outlined in Supplementary Figure S2.

### Rlp7 and L7 bind distinct sites within pre-ribosomal particles

**Figure 4.**
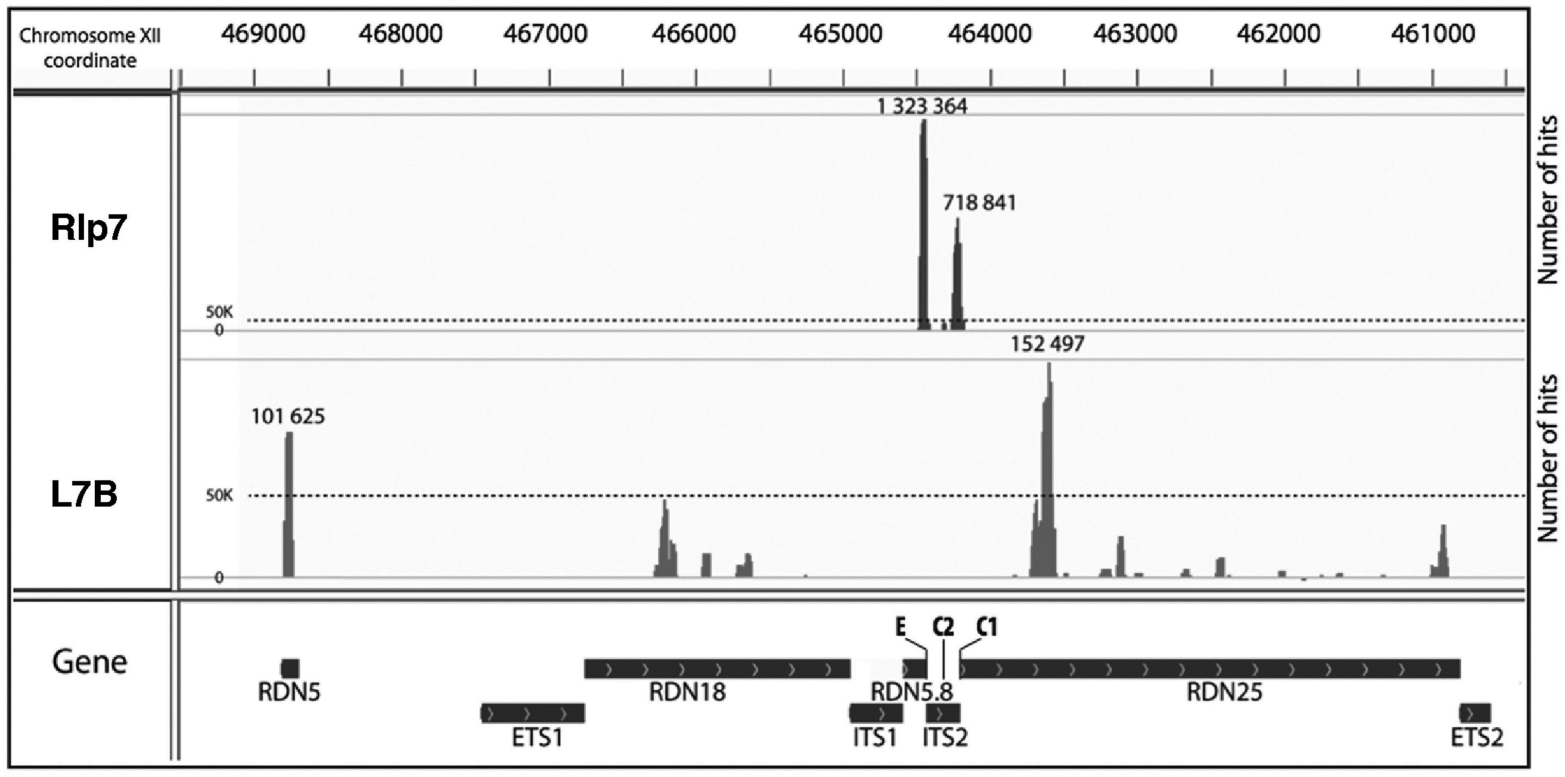
Identification of Rlp7 and L7 binding sites on pre- and mature rRNAs. The histograms, plotted using the Integrative Genomics Viewer software, display the sequences identified on CRAC analysis and the number of hits mapped to the rDNA. CRAC was performed with Rlp7-HTP (Rlp7) and L7B-HTP *rpl7AΔ* (L7B) cells. The maximum number of hits in the main peaks is shown.

**Figure 5.**
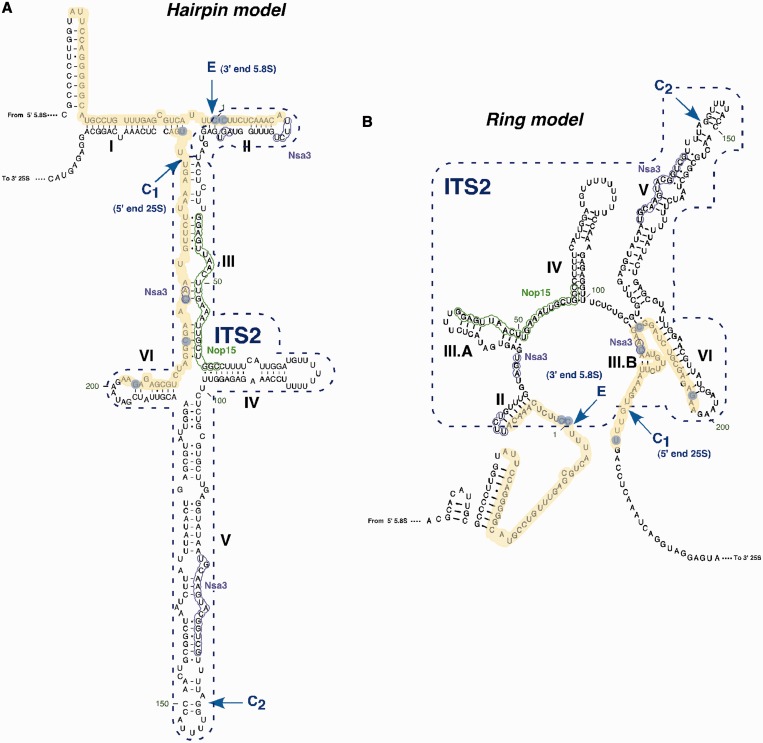
Localization of the CRAC interaction sites of Rlp7 with pre-rRNA sequences displayed on the ‘hairpin model’ (**A**) and on the ‘ring model’ (**B**) for yeast ITS2 secondary structure [for a reference, see (45)]. The CRAC sites are highlighted in yellow; blue circles indicate frequently mutated residues found in the experiments (see Supplementary Figure S8). The Nsa3 and Nop15 CRAC sites, as described in (7), are represented as purple and green, respectively. The location of the CRAC sites cleavage sites C_1_, C_2_ and E are also indicated.

L7 is a RNA binding protein (19) and due to its homology with L7, Rlp7 is also predicted to bind RNA. To find out whether Rlp7 and L7 share the same binding site on pre-60S ribosome, we attempted to identify their *in vivo* binding sites by using the CRAC method (9). The Rlp7-HTP strain did not show any growth phenotype at 30°C (Supplementary Figure S5). We also analysed L7B-HTP tagged strains, with or without the *RPL7A* endogenous copy, both presenting a phenotype consistent with the non-tagged corresponding strain (Supplementary Figure S5). We found that Rlp7-HTP directly and specifically contacts two regions in ITS2 (Figures 4 and 5, Supplementary Figure S7A, S8 and S9), whereas the non-ultraviolet cross-linked Rlp7-HTP protein did not significantly cross-link detectable rRNA (Supplementary Figure S7A). These two regions of ITS2 overlap the boundaries of 25S 5′ end and 5.8S 3′ end. Previous CRAC experiments with other A_3_ factors, Erb1, Nop7, Nop15 and Nsa3 revealed binding sites close and even overlapping these boundaries, consistent with the described collective role of these factors for processing of the 27SA_3_ pre-rRNA (6,7) (Figure 5 and Supplementary Figure S7A). Indeed, loss-of-function of the A_3_ factors (including Rlp7) leads to the accumulation of the 27SA_3_ pre-rRNA, reduced formation of 27SB_S_ relative to 27SB_L_ pre-rRNA and loss of cleavage at site C_2_ in ITS2 (6,7,20,21). Interestingly, the nucleotide substitutions analysis at specific positions in the sequence reads allowed us to precisely identify cross-link sites of Rlp7-HTP as nucleotides distributed in two groups, one adjacent to the pre-rRNA processing sites C_1_ and E that define the 5′ end and 3′ end of mature 25S and 5.8S rRNAs, respectively, and another at helix III at the 3′ end of ITS2, between nucleotides 200 and 225 (Figure 5 and Supplementary Figure S8).

**Figure 6.**
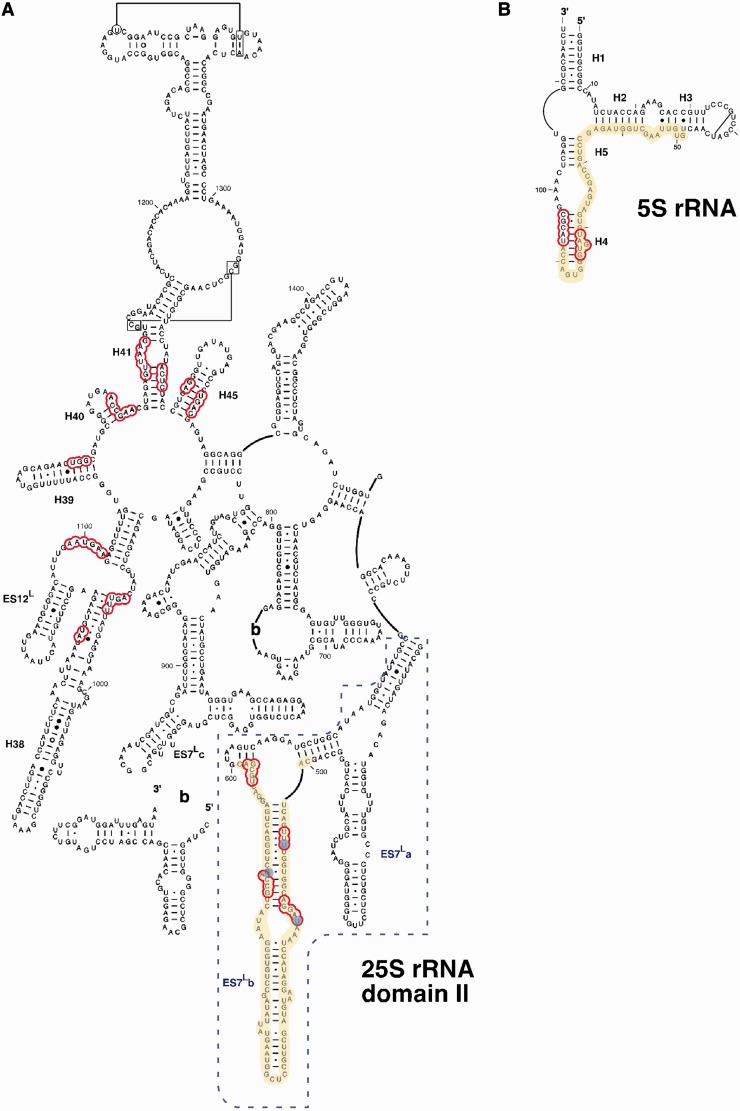
Localization of the CRAC interaction sites of L7 in the secondary structure of domain II of 25S rRNA (**A**) and 5S rRNA (**B**). The structures, residues and helix (H) numbers were taken from the Comparative RNA Web Site (46). The eukaryotic expansion segment ES7^L^ is labelled with a dash box. Red circles indicate rRNA residues situated in close proximity of L7 (closer than 5 Å) in the structure of yeast 60S r-subunit [PDB file 3U5H; (19)]. The CRAC sites are highlighted in yellow; blue circles indicate frequently mutated residues found in the experiments.

We also performed CRAC analyses with strains expressing HTP-tagged L7B either harbouring the wild-type *RPL7A* (LMA1551 strain) or the null *rpl7AΔ* allele (LMA1730 strain). The LMA1551 strain did not give a sufficient signal to get robust identification of binding sites; thus, we pursued the experiment only with the LMA1730 strain (Supplementary Figure S7B). We found that L7 mainly cross-linked to helix ES7^L^b in 25S rRNA, which corresponds to an expansion segment exclusive of eukaryotic 25S rRNA (19), and a long region of 5S rRNA over H2, H4 and H5 (Figures 4 and 6, Supplementary Figures S8 and S9). Base substitutions at positions U_508_, U_520_ and G_579_ in the 25S rRNA identified positions where L7 intimately contact 25S rRNA (Supplementary Figure S8). These results are in full agreement with the L7 interaction sites on domain II of 25S rRNA deduced from the large r-subunit crystal structure analysis (19). Additionally, minor cross-linking regions were identified along 25S rRNA and even at 18S rRNA that are at the threshold of what could be considered background (Figure 4).

Altogether, these findings clearly indicate that the Rlp7 and L7 binding sites are distinct in pre- and/or mature rRNA and are distant from each other, consistent with simultaneous binding of Rlp7 and L7 to pre-60S ribosomal particles without steric conflict.

## DISCUSSION

In this work, we report the unexpected discovery that both r-protein L7 and its related pre-60S factor Rlp7 bind to the same nuclear 60S precursors. Moreover, we show that despite their sequence and predicted structural similarity, the proteins bind to distinct sites on pre-rRNA.

Our SILAC and immunoprecipitation experiments indicate that Rlp7 mainly associates with early and medium nucle(ol)ar pre-60S r-particles and likely dissociates after cleavage of 27SB pre-rRNA, following exonucleolytic 7S pre-rRNA processing. These results complement those previously obtained by Woolford and co-workers (6). Strikingly, the most enriched and abundant pre-60S factors associated to Rlp7-TAP (Erb1, Nop7, Nsa3, Ytm1 and Nop15) are components of the A_3_ factors cluster whose loss-of-function leads to processing defects at A_3_ site with the subsequent accumulation of the 27SA_3_ precursor and depletion of its immediate 27SB_S_ and 7S pre-rRNA products [(6,7) and references therein]. The A_3_ factors are also intriguing because their association with pre-ribosomal particles appears to be interdependent (6). Moreover, A_3_ factors are required for proper assembly of four r-proteins (L17, L26, L35 and L37) that predominantly bind to 5.8S/25S rRNA domain I, which in turn enable cleavage of ITS2 at site C_2_ (6,14,18,47). Consistently, our CRAC analyses show that Rlp7 binds to ITS2 at a position adjacent with that of the 3′ end of mature 5.8S rRNA (site E) and the 5′ end of mature 25S rRNA (site C_1_). The Rlp7 binding sites on pre-rRNA partially overlap with those previously reported for the A_3_ factor Nsa3 and are close to those of other A_3_ factors (Nop12, Nop15, Erb1 and Nop7) (7). It has been shown that binding of Nsa3 to 27S pre-rRNAs is required to maintain a flexible and an open structure in ITS2 (the so-called ring conformation), which prevents the formation of the mutually exclusive hairpin structure of ITS2 (7). However, assuming that (i) Rlp7 contacts rRNA in a similar manner as L7 (see Supplementary Figure S9) and that (ii) one Rlp7 molecule simultaneously binds to the two Rlp7 binding sites, we propose that Rlp7 might preferentially interact with the hairpin structure of ITS2, thereby promoting the transition from the ring to the hairpin conformation of ITS2 that it is essentially required for ITS2 processing at site C_2_ (48). Consistently with this hypothesis, we identified the precise Rlp7 cross-linking sites by the presence of point mutations exactly at the site E and one nucleotide after the site C_1_. Sites E and C_1_ represent, respectively, precise positions where the exonuclease activities of the exosome and Rat1-Xrn1-Rrp17 stop at ITS2 (49–51). Whether Rlp7 acts blocking progression of these exonucleases beyond the processing sites is an attractive suggestion, which fits well with our results, but it needs further experimental evidence to be proven.

Our results also clearly indicate the early nucle(ol)ar assembly of L7 to ribosome precursors containing 27S pre-rRNAs. RNA and protein precipitation experiments strongly suggest that both Rlp7 and L7 co-exist in the same pre-ribosomal particles. Moreover, SILAC shows that L7 is as abundant as any other 60S r-protein in an Rlp7-TAP purified fraction. CRAC examination of the RNA binding sites of L7 in r-particles showed specific cross-links to helix ES7^L^b and 5S rRNA. These sites are far from the CRAC identified Rlp7 binding sites (see Supplementary Figure S9), therefore, suggesting mutually independent binding of both proteins with pre-ribosomal particles. Consistent with this, Woolford Jr and co-workers have demonstrated that levels of L7 in pre-60S r-particles were practically unaffected on depletion of Rlp7 (6). In the opposite experiment, depletion of L7 only slightly diminished levels of Rlp7 in pre-60S r-particles (15). Thus, Rlp7 is likely not the placeholder for the assembly of L7 r-protein; thus, L7 does not exchange with Rlp7 during 60S r-subunit biogenesis*.* This scenario is the opposite to that we have previously reported for the Mrt4-P0 pair of paralogues (23,42). In addition to Mrt4-P0 and Rlp7-L7, there are at least two other pairs of paralogues comprised by an r-like assembly factor and an r-protein in yeast, Imp3-S9 and Rlp24-L24. Whether the dynamics of these pairs during ribosome biogenesis resembles that of Mrt4-P0 or that of Rlp7-L7 evidently needs further investigation.

In conclusion, the hypothesis that a distinct *trans*-acting factor serves as a placeholder for its homologous r-protein is not applicable in all circumstances and, indeed, paralogues can co-exist within the same pre-ribosomal particles. Furthermore, genetic and biochemical experiments will be required to unravel the precise function of Rlp7 during ribosome assembly. Recently, it has been found that some archaeal r-proteins have more than one RNA binding site in ribosomes (52). These sites are structurally similar, which explains the promiscuous behaviour of these r-proteins. Whether the hairpin conformation of ITS2 resembles the structure of 25S rRNA domain II involved in the binding of L7 r-protein remains a challenging question for future studies. If this is the case, we will need to address then how proteins whose cores fold into apparently similar 3D structures could be specifically targeted to different locations in pre-ribosomal complexes.

## SUPPLEMENTARY DATA

Supplementary Data are available at NAR Online, including [53–58].

## FUNDING

Spanish Ministry of Science and Innovation and ERDF [BFU2010-15690 and FR2009-0102]; Andalusian Government [CVI-271 and P08-CVI-03508 to J.d.l.C.]; Agence Nationale de la Recherche [ANR-2011-BSV6-011-785 02]; EGIDE Picasso Programme (to M.F-R.); recipient of an FPI fellowship from the Andalusian Government (to R.B.). Funding for open access charge: Spanish Ministry of Science and Innovation and ERDF [BFU2010-15690 to J.d.l.C.].

*Conflict of interest statement*. None declared.

## Supplementary Material

Supplementary Data
