# Supplementary material for: Yeast ribosomal protein L7 and its homologue Rlp7 are simultaneously present at distinct sites on pre-60S ribosomal particles
Source: Nucleic Acids Res. 2013 Aug 13;41(20):9461–70. doi: 10.1093/nar/gkt726 (PMC3814368; doi:10.1093/nar/gkt726)
Supplement: Supplementary Data [file supp_41_20_9461__index.html]

Yeast ribosomal protein L7 and its homologue Rlp7 are simultaneously present at distinct sites on pre-60S ribosomal particles — Yeast ribosomal protein L7 and its homologue Rlp7 are simultaneously present at distinct sites on pre-60S ribosomal particles — Supplementary Data 

# Yeast ribosomal protein L7 and its homologue Rlp7 are simultaneously present at distinct sites on pre-60S ribosomal particles

## Supplementary Data

files

**Files in this Data Supplement:**

- Supplementary Data - pdf file
- Supplementary Data - xlsx file
