# Supplementary material for: Yeast ribosomal protein L7 and its homologue Rlp7 are simultaneously present at distinct sites on pre-60S ribosomal particles
Source: Nucleic Acids Res. 2013 Aug 13;41(20):9461–70. doi: 10.1093/nar/gkt726 (PMC3814368; doi:10.1093/nar/gkt726)
Supplement: Supplementary Data [file supp_gkt726_nar-01700-r-2013-File008.pdf]

SUPPLEMENTARY INFORMATION

SUPPLEMENTARY DATASETS AND TABLES

Supplementary Dataset S1. SILAC-based measurement for the quantified proteins in the experiments shown in **Figure 1**. The results of the two independent SILAC experiments were reproducible (Pearson correlation coefficient of 0.72, $p<2x10^{-16}$) with more than 30 pre-60S *trans*-acting factors being clearly enriched in the Rlp7-associated fraction in both experiments.

Supplementary Table S1. Yeast strains used in this study

Strain	Relevant genotype	Reference
BMA64-1B	*MATα leu2-3,112 trp1Δ ura3-1 ade2-1 his3-11,15*	(53)
LMA1496	As BMA64-1B but *RLP7-HTP::TRP1*	This work
LMA1551	As BMA64-1B but *RPL7B-HTP::TRP1*	This work
LMA1730	As BMA64-1B but *RPL7B-HTP::TRP1 RPL7A*::kanMX4	This work
BY4741	*MAT**a** his3Δ1 leu2Δ0 met15Δ0 ura3Δ0*	(54)
BY4742	*MATα his3Δ1 leu2Δ0 lys2Δ0 ura3Δ0*	(54)
Y04443	As BY4741 but *RPL7A::*kanMX4	Euroscarf
Y01094	As BY4741 but *RPL7B::*kanMX4	Euroscarf
LMA1738	As BY4742 but *RPL7B*::*HygroMX4* kanMX4::P_{GAL1}-*RPL7A*	This work
LMA1830	As BY4742 but *RPL7B*::*HygroMX4* kanMX4::P_{GAL1}-*RPL7A RLP7-TAP::HIS3MX6*	This work
LMA1821	As BY4742 but *RLP7-TAP::HIS3MX6*	This work
LMA1779	As BY4741 but *RPL7B-TAP::*HIS3MX4	Open Biosystems
Y1272	As BY4741 but *RPL7B-GFP::*HIS3MX4	Invitrogen
Y1875	As BY4741 but *RPL3-GFP::*HIS3MX4	Invitrogen
Y25330	*MAT**a**/MATα his3Δ1/his3Δ1 leu2Δ0/leu2Δ0 lys2Δ0/LYS2 met15Δ0/MET15 ura3Δ0/ura3Δ0 RLP7/RLP7::*kanMX4	Euroscarf
RBY278	As BY4741 but *RLP7::*kanMX4 [pGAD3-RLP7]	This work
RBY279	As BY4741 but *RPL7B-TAP*::HIS3MX4 *RLP7*::kanMX4 [pGAD3-RLP7]	This work
RBY280	As BY4741 but *RPL7B-TAP::*HIS3MX4 *RLP7*::kanMX4 [YCplac111-RLP7-HA]	This work
RBY281	As BY4741 but *RLP7*::kanMX4 [YCplac111-RLP7-HA]	This work

Supplementary Table S2. Plasmids used in this study

Plasmid	Features	Reference
pGAD3-RLP7[(a)]	*2μ LEU2 GAL::RLP7*	(20)
pHAC111	*CEN LEU2 HA* tag	(27)
YCplac111-RLP7-HA	*CEN LEU2 RLP7-HA*	This study
pRS316-NOP1-RFP-RPL25-GFP[(b)]	*CEN URA3 NOP1-mRFP RPL25-yEGFP*	(55)
pRS316-GAL-NMD3FL[(c)]	*CEN URA3 GAL::NMD3*	(56)
pRS316-GAL-NMD3Δ100[(c)]	*CEN URA3 GAL::NMD3Δ100*	(56)

(a) Gift of S. Baserga

(b) Gift of J. Bassler and E. Hurt

(c) Gifts of A. Jacobson

Supplementary Table S3. Oligonucleotides used in this study[(a)]

Name	5'-3' Sequence
Probe a (5' A_0)	GGTCTCTCTGCTGCCGG
Probe b (18S)	CATGGCTTAATCTTTGAGAC
Probe c (3-D/A_2)	GACTCTCCATCTCTTGTCTTCTTG
Probe d (A_2/A_3)	TGTTACCTCTGGGCCC
Probe e (5.8S)	TTTCGCTGCGTTCTTCATC
Probe f (E/C_2)	GGCCAGCAATTTCAAGTTA
Probe g (C_1/C_2)	GAACATTGTTCGCCTAGA
Probe h (25S)	CTCCGCTTATTGATATGC
Probe 5S	GGTCACCCACTACACTACTCGG

(a) All primers used to verify strains, construct plasmids or perform the CRAC experiments are not shown in this table and are available upon request.

LEGENDS TO SUPPLEMENTARY FIGURES

Supplementary Figure S1. Pre-rRNA processing in *S. cerevisiae*. (**A**) Structure of a rDNA repeat unit. Each unit contains a large element encoding 18S, 5.8S and 25S rRNAs, which is transcribed by RNA polymerase I, and a short element encoding 5S rRNA, which is transcribed by RNA polymerase III. Non-transcribed, external and internal spacers (NTS, ETS and ITS, respectively) are indicated. The sequences for the mature rRNA species are shown as bars and the spacers as lines (NTSs are shown thinner than ETSs or ITSs). Transcription start sites are shown as red arrows. Processing sites and various probes used are indicated. (**B**) Pre-rRNA processing pathway. RNA pol I transcript can undergo either post- or co-transcriptional processing. Cleavage and trimming reactions are indicated. Note that, following either post- or co-transcriptional processing, 20S pre-rRNA is exported to the cytoplasm where it undergoes dimethylation ($m^2{}_6A$) and further cleavage at site D to generate the mature 18S rRNA. The $27SA_2$ precursor is processed by two alternative pathways that both lead to the formation of mature 5.8S and 25S rRNAs. Around 85% of $27SA_2$ pre-rRNA is cleaved at the site A_3 and then exonucleolytically processed to yield the $27SB_S$ pre-rRNA. The remaining 15% of $27SA_2$ pre-rRNA is directly cleaved at site B_{1L}, producing the $27SB_L$ pre-rRNA. The subsequent ITS2 processing of both 27SB species appears to be identical. Cleavage at site C_2 releases 25.5S pre-rRNA and the long and short forms of 7S pre-rRNA. Processing of 25.5S pre-rRNA to mature 25S rRNA requires 5'-3' exonucleolytic processing. Maturation of 7S pre-rRNAs to mature 5.8S rRNAs requires 3'-5' exonucleolytic processing. Note that processing of 6S pre-rRNAs occurs in the cytoplasm. For further description of the yeast pre-rRNA processing pathway, see reference (1).

Supplementary Figure S2. Model for the assembly position of r-protein L7. The cartoon shows a series of distinct intermediates during the 60S r-subunit synthesis. These are termed, according to their position in the pathway, early 0 (E_0), early 1 (E_1), early 2 (E_2) and medium (M) 66S pre-ribosomal particles and late (L) and cytoplasmic (C) pre-60S r-particles. All these intermediates are defined by the purification of complexes associated with TAP-tagged versions of selected r-subunit biogenesis factors (for reviews see, (4,57)). The predominant pre-rRNAs associated with the different particles are indicated. The data presented in this study indicate that Rlp7 and L7 bind to different positions during the maturation pathway of 60S r-subunits. Thus, Rlp7 mainly loads on ITS2 sequences within early pre-60S r-particles in the nucleolus and dissociates from intermediate particles

concomitantly to the processing of 7S pre-rRNA to mature 5.8S rRNA. Concurrently, r-protein L7 assembles in the nucleolus with 25S and 5S rRNA sequences into early pre-60S r-particles. Rlp7, blue dot; L7, red dot; nucleolus, grey rectangle; pre-ribosomal particles and mature r-subunits, ovals; nuclear envelope, rods.

Supplementary Figure S3. Rlp7 and L7 are paralogous proteins. Comparison of the amino acid sequence of Rlp7 and the L7A and L7B r-proteins from *S. cerevisiae*. Proteins were aligned using ClustalW2 at EMBL-EBI (www.ebi.ac.uk/Tools/clustalw2/).

Supplementary Figure S4. Predicted structure of Rlp7. (**A**) Structure of r-protein L7 as shown in atomic model for the crystal structure of the yeast 60S r-subunit described in Ben-Shem *et al.* (Protein Data Bank 3U5I; L7 is chain F (19)). Note that the N-terminal end of L7 could not be modelled and the structure starts at Thr_{22}. (**B**) Model of the Rlp7 protein structure generated with the Modeller program using different L7 structures as template. Note that the N-terminal end of the protein could not be modelled and the structure starts at Lys_{84}. We could not found homologous sequences for this N-terminal stretch in the databases. Moreover, we found that this region shows a high probability of being disordered according to IUPrep analysis (58) (**C**) Superimposition of L7 and the homology model of Rlp7. Images were generated with the UCSF Chimera program (40).

Supplementary Figure S5. Growth test of different strains used in this study. (**A**) Strains BMA64-1B (*Wild-type*), LMA1496 (*RLP7-HTP*), LMA1551 (*RPL7B-HTP*) and LMA1730 (*RPL7-HTP rpl7AΔ*). (**B**) Strains BY4741 (*Wild-type*), Y04443 (*rpl7AΔ*) and Y01094 (*rpl7BΔ*). All strains were grown in liquid YPD medium and diluted to an OD_{600} of 0.05. A 10-fold series of dilutions was spotted on YPD plates and incubated at 30 ºC for 3 days.

Supplementary Figure S6. L7 assembles within the nucleolus. Localisation of L3-GFP and L7B-GFP upon induction of the *NMD3* dominant-negative allele, *NMD3Δ100.* Cells from Y1272 (*RPL7B-GFP*) and Y1875 (*RPL3-GFP*) strains were transformed with the pRS316-GAL-NMD3Δ100 plasmid and transformants were grown in the presence of raffinose (Raf). Galactose (Gal) was then added to fully induce the Nmd3Δ100 protein. The GFP signal was inspected by fluorescence microscopy after 24 h. Arrows point to the nucleus.

Supplementary Figure S7. Autoradiogram of CRAC gels after transfer to nitrocellulose

membranes. Eluate 1 and 2 correspond to two consecutive eluates from the nickel columns (E1 and E2). (**A**) CRAC was performed with LMA1496 strain expressing RLP7-HTP plus or minus UV cross-linking (**B**) CRAC was performed with L7B-HTP tagged samples from the LMA1551 or LMA1730 strains, which express L7B-HTP in a *RPL7A* or a *rpl7AΔ* background, respectively, and plus or minus UV cross-linking. Boxes indicate the regions of the membranes excised to recover RNAs.

Supplementary Figure S8. Identification of the putative binding sites of Rlp7 and L7 on pre- and mature rRNAs. The Rlp7 and L7 minimal identified sequences of the major peaks after CRAC analysis are shown. Nucleotides coloured in red correspond to ITS2 and those in black to mature rRNAs. Hot spots of base mutations are numbered (1 to 9). Each position corresponds to a crosslink point; the percentage of mutation at a particular position is indicated.

Supplementary Figure S9. Localisation of L7 in the three-dimensional structure of the yeast 60S r-subunit. (**A**) Position of L7 relative to that of r-proteins P0, L5 and L24 and rRNAs 5.8S and 5S in the 60S r-subunit. The image was generated with the UCSF Chimera program (40) using the 60S r-subunit structures described in Ben-Shem *et al.* (3U5I and 3U5H (19)). L7 and Rlp7 CRAC binding regions are depicted. The location of the Erb1 and Nop7 CRAC sites, as described in (7), is also represented. (**B**) Close-up view of the position of L7 (red) in the context of its binding sites as identified by our CRAC results and the $ES7^{L}b$ segment. $ES7^{L}b$, $ES12^{L}$ from 25S rRNA are coloured in pink and helices H2 and H5 from 5S rRNA in green.

SUPPLEMENTARY FIGURES

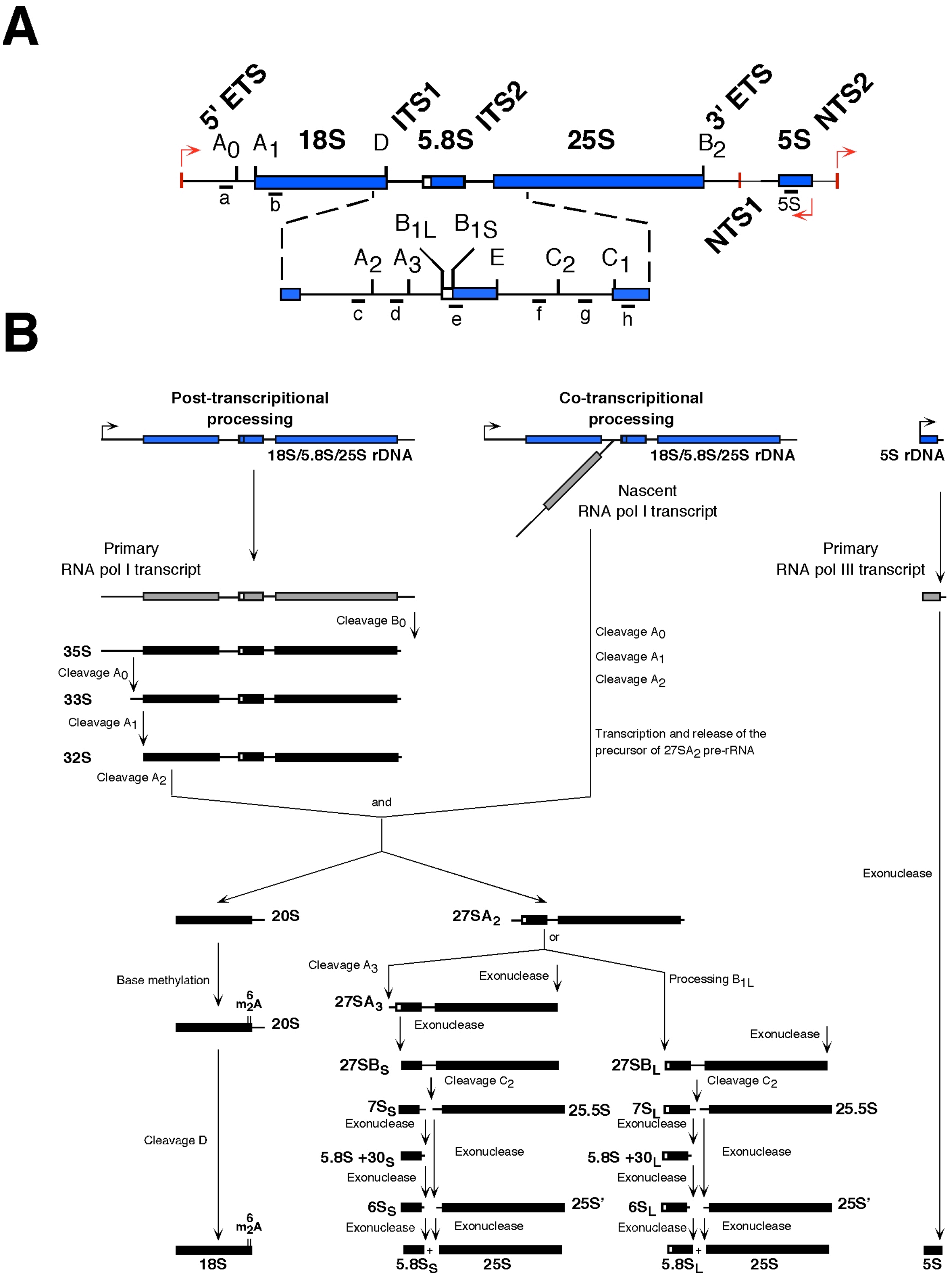

Supplementary Figure S1

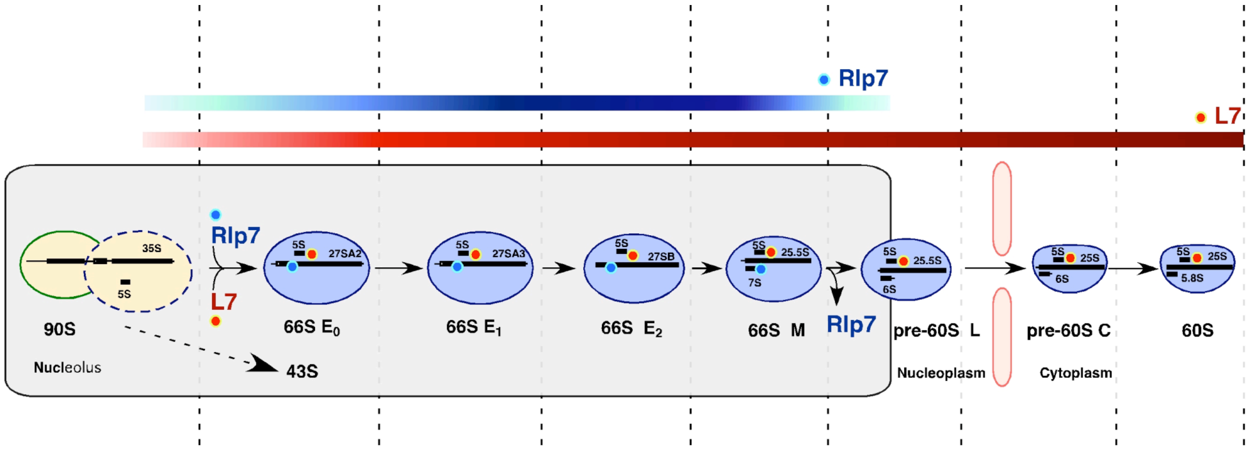

Supplementary Figure S2

```
L7A             -------MAAEKILTPESQLKKSK------------AQQKTAEQVAAER----------- 30
L7B             -------MSTEKILTPESQLKKTK------------AQQKTAEQIAAER----------- 30
Rlp7            MSSTQDSKAQTLNSNPEILLRKRRNADRTRIERQELAKKKREEQIKKKRSNKNKFVRAES 60
                        :     .**  *:* :            *::*  **:  :*

L7A             ------AARKAANKEKRAIILERNAAYQKEYETAERNIIQAKRDAKAAGS---------- 74
L7B             ------AARKAANKEKRAIILERNAAYQKEYETAERNIIQAKRDAKAAGS---------- 74
Rlp7            IVAKTLATSREKERIKRVSILEDKKAKNETQHIASGKDFILKITEKANGAEENSVDLEET 120
                      *: :  :: **. *** : * ::  . *. : :   *   ** *:

L7A             ------------YYVEAQHKLVFVVRIKG--INKIPPKPRKVLQLLRLTRINSGTFVKVT 120
L7B             ------------YYVEAQHKLVFVVRIKG--INKIPPKPRKVLQLLRLTRINSGTFVKVT 120
Rlp7            EEEEDDGLIREKTTYDGKPALLFIVRVRGPLAVNIPNKAFKILSLLRLVETNTGVFVKLT 180
                               :.:  *:*:**::*    :** *. *:*.****.. *:*.***:*

L7A             KATLELLKLIEPYVAYGYPSYSTIRQLVYKRG------FGKINKQRVPLSDNAIIEANLG 174
L7B             KATLELLKLIEPYVAYGYPSYSTIRQLVYKRG------FGKINKQRVPLSDNAIIEANLG 174
Rlp7            KNVYPLLKVIAPYVVIGKPSLSSIRSLIQKRGRIIYKGENEAEPHEIVLNDNNIVEEQLG 240
                * .  ***:* ***. * ** *:**.*: ***       .: : :.: *.** *:* :**

L7A             KYGILSIDDLIHEIITVGPHFKQANNFLWPFKLSNPSGGWGVPRKFKHFIQGGSFGNREE 234
L7B             KYGILSIDDLIHEIITVGPHFKQANNFLWPFKLSNPSGGWGVPRKFKHFIQGGSFGNREE 234
Rlp7            DHGIICVEDIIHEIATMGESFSVCNFFLQPFKLNREVSGFGSLNRLRKIKQREAESRTRQ 300
                .:**:.::*:**** *:*  *. .* ** ****..  .*:*  .::::: *  : .. .:

L7A             FINKLVKSMN------------ 244
L7B             FINKLVKAMN------------ 244
Rlp7            FSNAATAPVIEVDIDSLLAKLN 322
```

Supplementary Figure S3

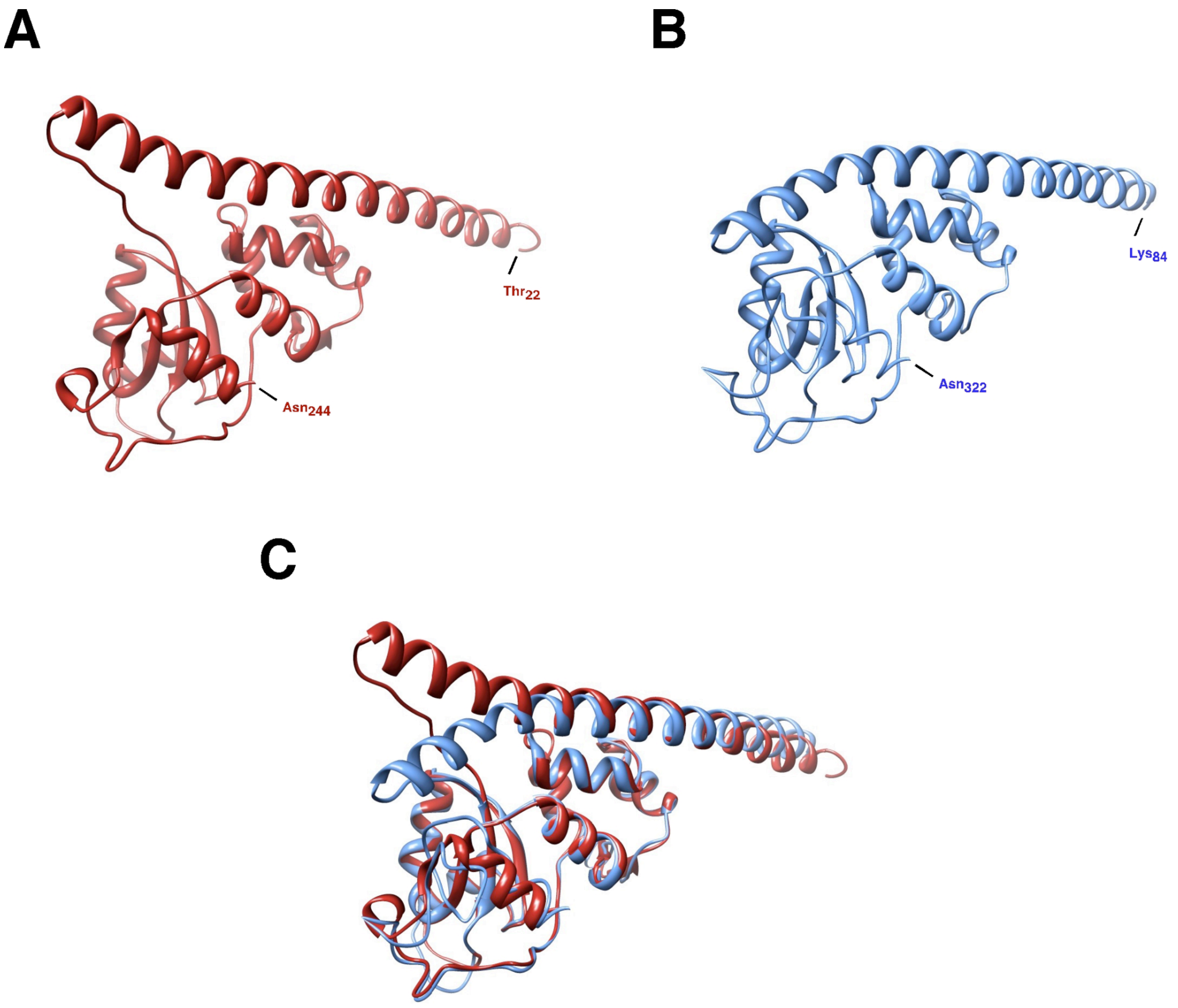

Supplementary Figure S4

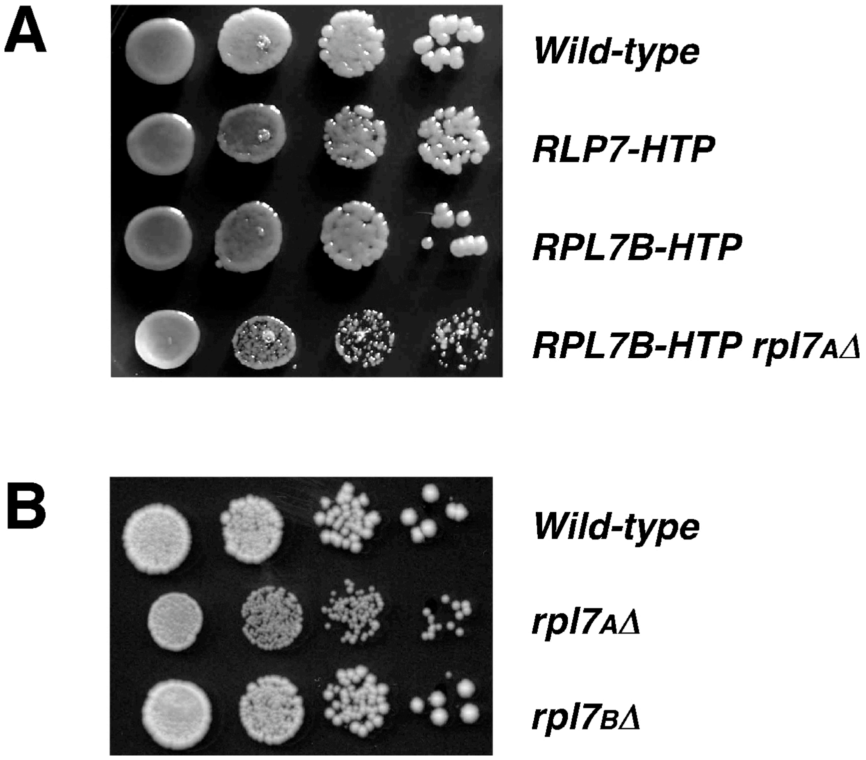

Supplementary Figure S5

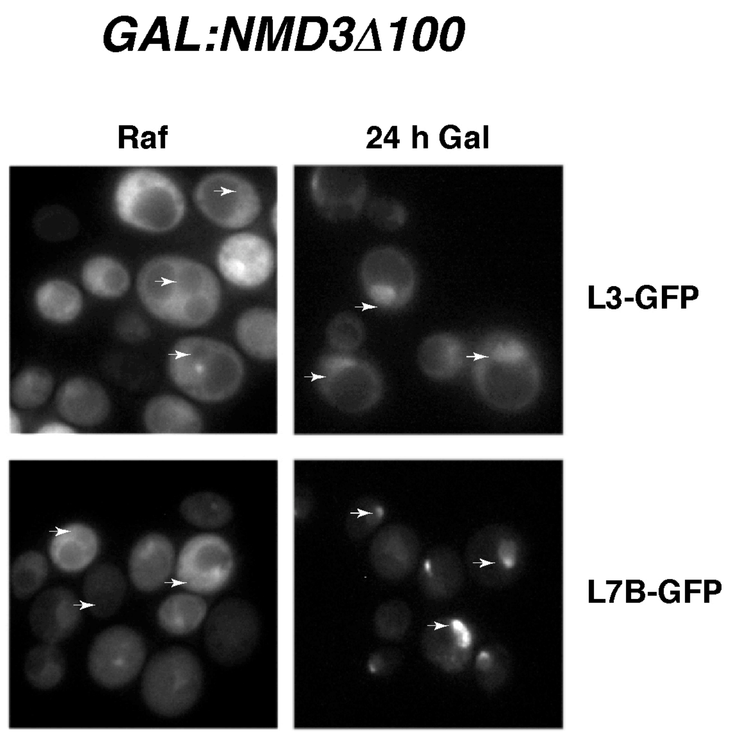

Supplementary Figure S6

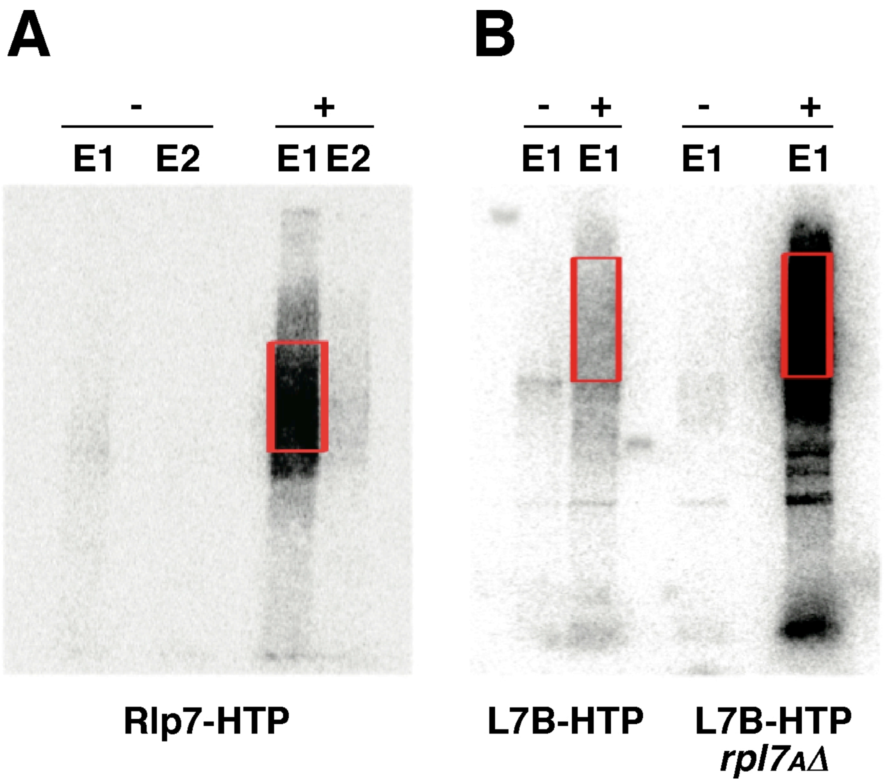

Supplementary Figure S7

Rlp7 crosslink points

• **Sequence in pre-rRNA (ITS2-25S):**

1 2 3 4

AA**g**AGAGCGUCUAGG**c**GAA**c**AAUGUUCUUAAAGUU**u**GA

(1) G to A (23%); **(2)** C to G (22%), A (1%); **(3)** C to A (22%), A (1%); **(4)** U to A (20%)

• **Sequence in pre-rRNA (5.8S-ITS2):**

56

AUUCCAGGGGGCAUGCCUGUUUGAGCGUCAUUU**cc**UUCUCAAACAA

(5) C to U (30%); **(6)** C to U (37%)

L7 crosslink points

• **Sequence in 25S rRNA (region 500 to 600 in 25S rRNA):**

7 8

CAUCAGUU**u**UGGUGGCAGGA**u**AAAUCCAUAGGAAUGUAGCUUGCCUCGGUAAGU

9

AUUAUAGCCUGUGGGAAUACUGCCA**g**CUGGGACUGAGGACUGCGACG

(7) U to C (21%); **(8)** U to A (31%), C (6%); **(9)** G to A (21%), G (3%)

• **Sequence in 5S rRNA:**

GUAGUUAAGCUGGUAAGAGCCUGACCGAGUAGUGUAGUGGGUGACCA

Supplementary Figure S8

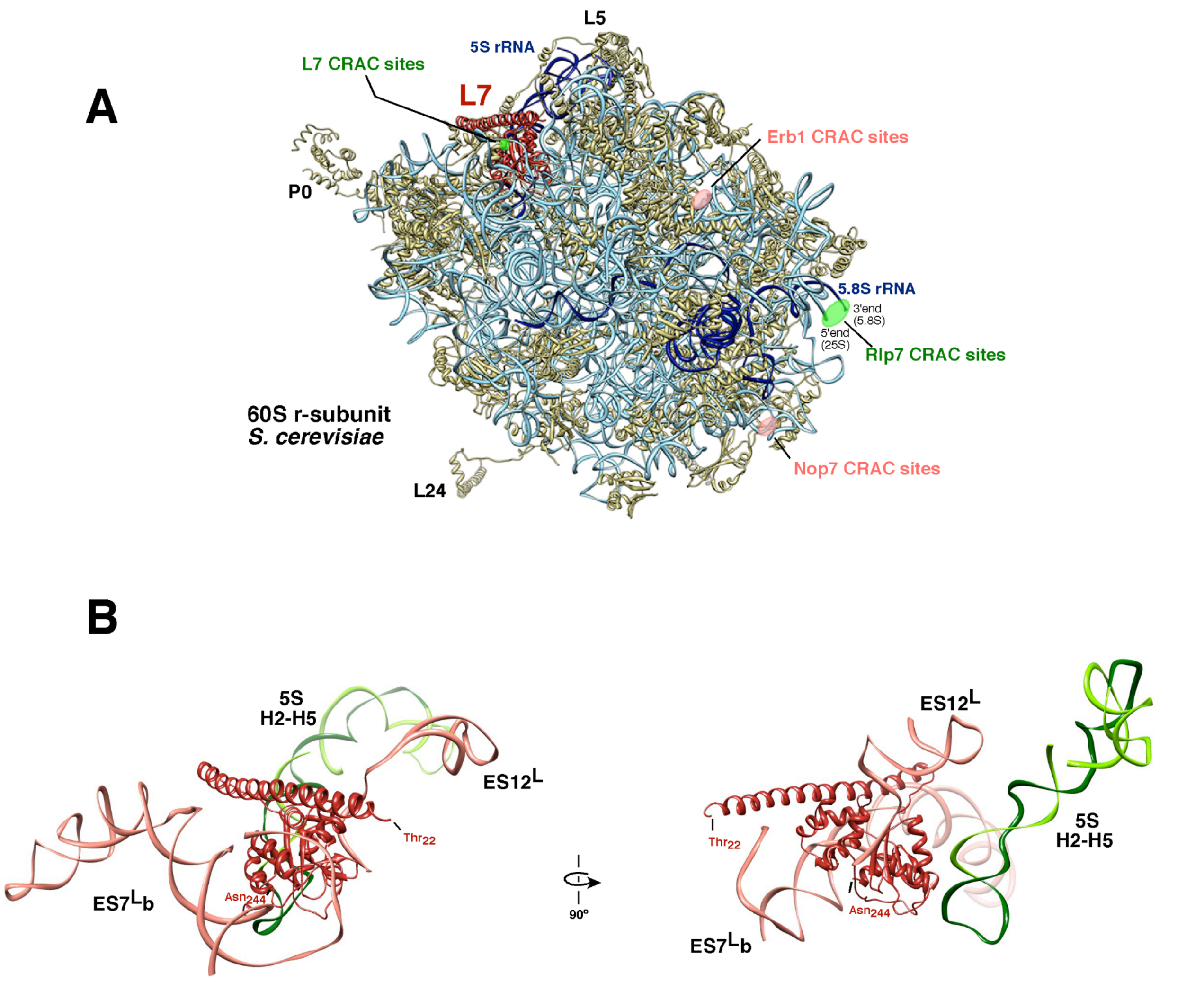

Supplementary Figure S9
